# Fabrication of Al_2_O_3_ Nano-Structure Functional Film on a Cellulose Insulation Polymer Surface and Its Space Charge Suppression Effect

**DOI:** 10.3390/polym9100502

**Published:** 2017-10-12

**Authors:** Jian Hao, Yanqing Li, Ruijin Liao, Guoyong Liu, Qiang Liao, Chao Tang

**Affiliations:** 1 The State Key Laboratory of Power Transmission Equipment & System Security and New Technology, Chongqing University, Chongqing 400044, China; cqu0926@126.com (Y.L.); rjliao@cqu.edu.cn (R.L.); 2 College of Material Science and Engineering, Chongqing University, Chongqing 400044, China; 20160913152@cqu.edu.cn; 3 College of Power Engineering, Chongqing University, Chongqing 400044, China; lqzx@cqu.edu.cn; 4 College of Engineering and Technology, Southwest University, Chongqing 400715, China; tangchao_1981@163.com

**Keywords:** nano-structure Al_2_O_3_ film, magnetron sputtering, cellulose insulation pressboard, space charge, suppression effect, HVDC transformer

## Abstract

Cellulose insulation polymer (paper/pressboard) has been widely used in high voltage direct current (HVDC) transformers. One of the most challenging issues in the insulation material used for HVDC equipment is the space charge accumulation. Effective ways to suppress the space charge injection/accumulation in insulation material is currently a popular research topic. In this study, an aluminium oxide functional film was deposited on a cellulose insulation pressboard surface using reactive radio frequency (RF) magnetron sputtering. The sputtered thin film was characterized by the scanning electron microscopy/energy dispersive spectrometer (SEM/EDS), X-ray photoelectron spectroscopy (XPS), and X-ray diffraction (XRD). The influence of the deposited functional film on the dielectric properties and the space charge injection/accumulation behaviour was investigated. A preliminary exploration of the space charge suppression effect is discussed. SEM/EDS, XPS, and XRD results show that the nano-structured Al_2_O_3_ film with amorphous phase was successfully fabricated onto the fibre surface. The cellulose insulation pressboard surface sputtered by Al_2_O_3_ film has lower permittivity, conductivity, and dissipation factor values in the lower frequency (<10^3^ Hz) region. The oil-impregnated sputtered pressboard presents an apparent space-charge suppression effect. Compared with the pressboard sputtered with Al_2_O_3_ film for 90 min, the pressboard sputtered with Al_2_O_3_ film for 60 min had a better space charge suppression effect. Ultra-small Al_2_O_3_ particles (<10 nm) grew on the surface of the larger nanoparticles. The nano-structured Al_2_O_3_ film sputtered on the fibre surface could act as a functional barrier layer for suppression of the charge injection and accumulation. This study offers a new perspective in favour of the application of insulation pressboard with a nano-structured function surface against space charge injection/accumulation in HVDC equipment.

## 1. Introduction

Pursuing high efficiency in electric power transmission and renewable energy has led to rapid developments in high voltage HVDC transmission systems. One of the most challenging issues in HVDC insulation material development and insulation structure design is the space charge accumulation within the insulation material [1]. The formation of space charge in the insulation system can result in a distortion of the electric field distribution, i.e., an enhanced electric field in one region, and a reduced electric field in another. This leads to material degradation in the high electric field region, and affects system reliability [2,3,4,5]. Therefore, effective ways to suppress space charge accumulation have been considered the key foundation in designing and ensuring the safety of polymeric HVDC insulation material [1].

Most of the attempts at reducing the space charge involve modifying the insulation material via the dispersion of nano-fillers into the polymer bulk [6,7,8,9,10,11,12,13,14,15,16,17,18]. The nanocomposite was first reported by Lewis in 1994 [6], and since then, it has been proved to be very effective in suppressing space charge in polymers [7,8,9,10,11,12,13,14,15,16,17,18]. For the low density polyethylene (LDPE) used as direct current (DC) cable insulation, a reduction in space charge accumulation in the LDPE can be achieved by incorporating a small percentage of inorganic nanoparticles (e.g., Al_2_O_3_, ZnO, MgO and SiO_2_) [8,9,10,11]. For the epoxy resin coils that are used in motors, generators, and gas-insulated switchgears, the epoxy nanocomposite (SiO_2_, Al_2_O_3_, MgO) shows weaker space charge accumulation [12,13,14]. For the cellulose insulation paper that is widely used in HVDC transformers, the dielectric properties of nano-Al_2_O_3_ and nano-SiO_2_ doped paper were studied [15,16,17,18]. The electrical properties of nano-Al_2_O_3_ and nano-SiO_2_ doped papers are better than those of the conventional paper, in particular, there is less space charge accumulation in the bulk. A sandwich-structured nano-composite (nano-LDPE/LDPE/nano-LDPE), which is not exactly the same as the above method, was also reported [8,19]. Unlike the above nanocomposites which are composed of one layer, the sandwich-structured nanocomposites (Al_2_O_3_–LDPE/LDPE/Al_2_O_3_–LDPE) are composed of three layers. The middle layer is neat LDPE, and the other two layers is Al_2_O_3_–LDPE nanocomposite which is made by the dispersion of nano-Al_2_O_3_ into the LDPE. The total charge accumulated in the sandwich-structured nanocomposite (nano-LDPE/LDPE/nano-LDPE) is less than that in neat LDPE and nano-LDPE nanocomposites, which indicates that the elaborate structural design can inhibit the space charge.

Nanoparticles shows a good space charge suppression effect, owing to their small size and large specific surface area [6,7]. Therefore, in addition to adding nano-fillers to the material, it is worth investigating the fabrication of a special functional nano-structure film on the surface of the insulating material, which could provide an effective suppression function for space charge injection and accumulation, compared with that of a material with nanoparticles filled in the bulk. In the field of material science, many methods have been used to prepare nano-/micro-structure functional films, including magnetron sputtering, ion beam sputtering, sol-gel, pulsed laser deposition, physical vapour deposition, and chemical solution deposition [20,21,22,23,24,25,26,27,28]. Compared with other methods, magnetron sputtering has many advantages, such as easier thickness control, better film compactness, and better adhesion of film and substrate [29,30,31,32,33,34,35]. Many researchers successfully fabricated functional films (such as ZnO, TiO_2_/SiO_2_, Al_2_O_3_) by radio magnetron sputtering on the metal and glass material [29,30,31,32,33,34,35]. However, no research has reported on the fabrication of nano/micro-structure functional films on cellulose insulation material to inhibit charge injection. 

In this study, considering the Al_2_O_3_ is a well-known insulator that has good mechanical, thermal, and chemical stability [15,16,18] and is frequently used as a coating material and nano-filler for insulation paper [15,16,18,33,34,35], an Al_2_O_3_ functional thin film was deposited on cellulose insulation pressboard by using reactive RF magnetron sputtering at room temperature. The physical and chemical characterisation of the as-prepared functional film was presented. The sputtered film attached on the surface of the insulation pressboard is expected to be nano–structure, which means the particles’ size is in nanometer level. The influence of the Al_2_O_3_ function thin film on the dielectric properties and the space charge behaviour of the sputtered pressboard were investigated. This research provides a new point of view for restraining space charge injection in cellulose insulation material.

## 2. Materials and Methods

### 2.1. Materials and Sample Preparation

**Figure 1:**
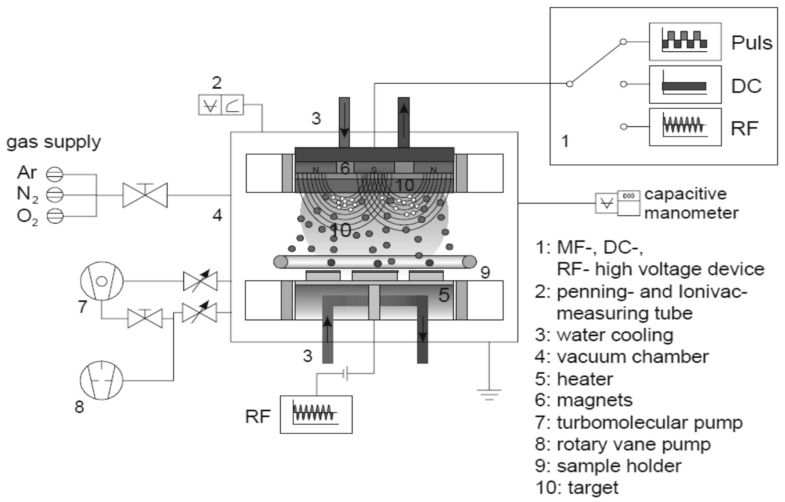
Principle of the reactive radio frequency (RF) magnetron sputtering [33,34,35].

Cellulose insulation pressboard (thickness 0.5 mm) was used for reactive RF magnetron sputtering provided by the NARI Borui transformer factory, Chongqing, China. The insulation pressboard substrates were cut into 15 cm × 10 cm pieces. The JPGF-480 reactive RF magnetron sputtering device (Beijing Instrument Factory, Beijing, China) at 13.56 MHz was used in this experiment. The principle of reactive RF magnetron sputtering is shown in Figure 1 [33,34,35]. RF sputtering works well to produce oxide films. For the deposition of Al_2_O_3_ film, an aluminium target (diameter 61.5 mm, thickness 5 mm) of 99.999% purity was sputtered. The distance between the target and the substrate sample was 10 cm. The vacuum chamber was pumped down to a base pressure of 4.0 × 10^−3^ Pa before sputtering. Deposition processes were performed by using 110 W of forward power. Argon (Chongqing Hong Hao Gas Co., Ltd., Chongqing, China) was used as the working gas, with a constant pressure of 1.5 Pa. Oxygen (Chongqing Hong Hao Gas Co., Ltd., Chongqing, China) was the reactive gas and had a flow of 20 sccm. The total pressure was constant throughout the sputtering procedure. The deposition mode was static, and the deposition time were 60 and 90 min at room temperature (28 °C).

### 2.2. Characterisation Methods

The film characterisation focused primarily on the structural properties, dielectric properties and the space charge suppression effect. For a detailed investigation of the microstructural changes, a scanning electron microscopy/energy dispersive spectrometer (SEM/EDS, JSM-7800F, JEOL, Tokyo, Japan) was employed to characterize the surface morphology and relative composition of the deposited film. X-ray photoelectron spectroscopy (XPS, Thermo escalab 250Xi, Waltham, MA, USA) was performed with Al Kα X-ray source to characterize the chemical binding state and composition of the deposition film. The XPS measurement was performed without argon etching. The XPS spectra were recorded in the fixed analyzer transmission mode with pass energy of 20 eV and resolution 0.1 eV. The deviation caused by the charging effect was calibrated using adventitious carbon referencing (C 1s, 284.6 eV). The X-ray diffraction (XRD) patterns were obtained by an X-ray diffractometer (PANalytical Empyrea, Almelo, The Netherlands) using Cu Ka (λ = 0.154056 nm) radiation at a fixed incident angle of 2°. The SEM/EDS, XPS, and XRD measurements were performed three days after the completion of sputtering. The samples were packed well in a plastic box, without contacting any material.

**Figure 2:**
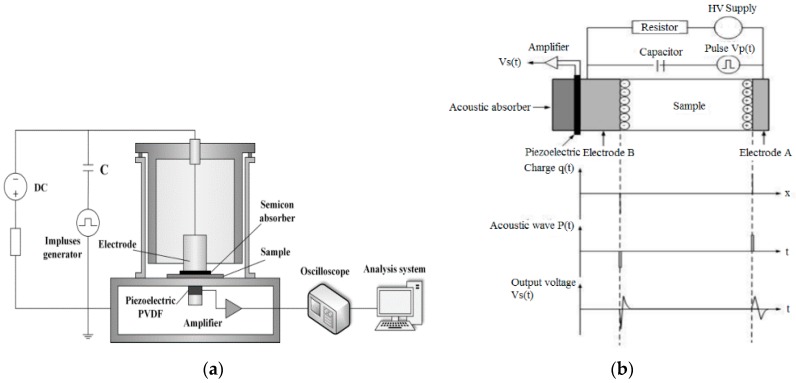
Schematic principle PEA method [36] (**a**) experiment setup; (**b**) PEA principle.

Frequency dielectric spectroscopy (FDS) was performed using Novocontrol Concept 80 Broadband Dielectric Spectroscopy equipment (Novocontrol GmbH, Montabaur, Germany) from 10^−2^–10^6^ Hz at room temperature. Researchers have used the pulsed electro-acoustic (PEA) method to measure the space charge in solid dielectrics. The principle of the PEA method can be seen in many studies [2,4,36], in brief, it consists of detecting the acoustic waves generated by internal charges under the Coulomb force of a pulsed electric field. The waves are detected by an external piezoelectric transducer, which converts the acoustic signal into an electrical signal. Then, the internal charge density is deduced by signal processing and mathematic treatment. The PEA principle is schematically represented in Figure 2 [36], where *q*(*t*) is the electric charge distributed in the sample, *P*(*t*) is the acoustic pressure wave as a function of time, the shape of *P*(*t*) is the same as the pulse electric field, and *Vs*(*t*) is the transducer output as a voltage signal. The PEA system (Shanghai Jiaotong University, Shanghai, China) has a pulse voltage of 600 V and a width of 5 ns. The bottom electrode is made of a 10 mm thick aluminium plate, and the top electrode is the semiconducting polymer film. 

Before the FDS and space charge measurements, the untreated and treated pressboard sputtered for 60 and for 90 min were put into three glass bottles. All of the bottles and their samples were put into a vacuum box and dried at 90 °C for 24 h. Then, the temperature of the vacuum box was adjusted to 40 °C. New mineral oil was then infused into the vacuum box. The vacuum box was left for 48 h for pressboard impregnation at 40 °C before being cooled to room temperature. After impregnation, the moisture content of the oil-impregnated pressboard was 1.35%, according to the Karl Fischer Titration method. The PEA measurement testing temperature was room temperature. The pressboard surface on which only one side had a sputtered aluminium oxide thin film was attached to the cathode when the measurement was performed. The threshold electric field in oil impregnated insulation paper was usually about 12 kV/mm [2,4]. When the applied electric filed was higher than 12 kV/mm, charge injection occurred. In this experiment, the DC electric filed was 15 kV/mm.

## 3. Results

### 3.1. SEM/EDS of the Sputtered Film

**Figure 3:**
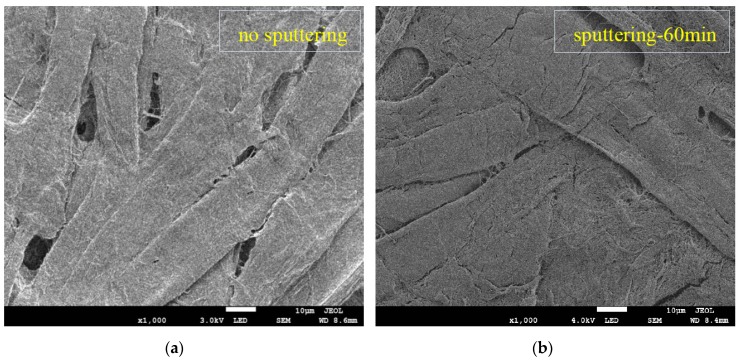

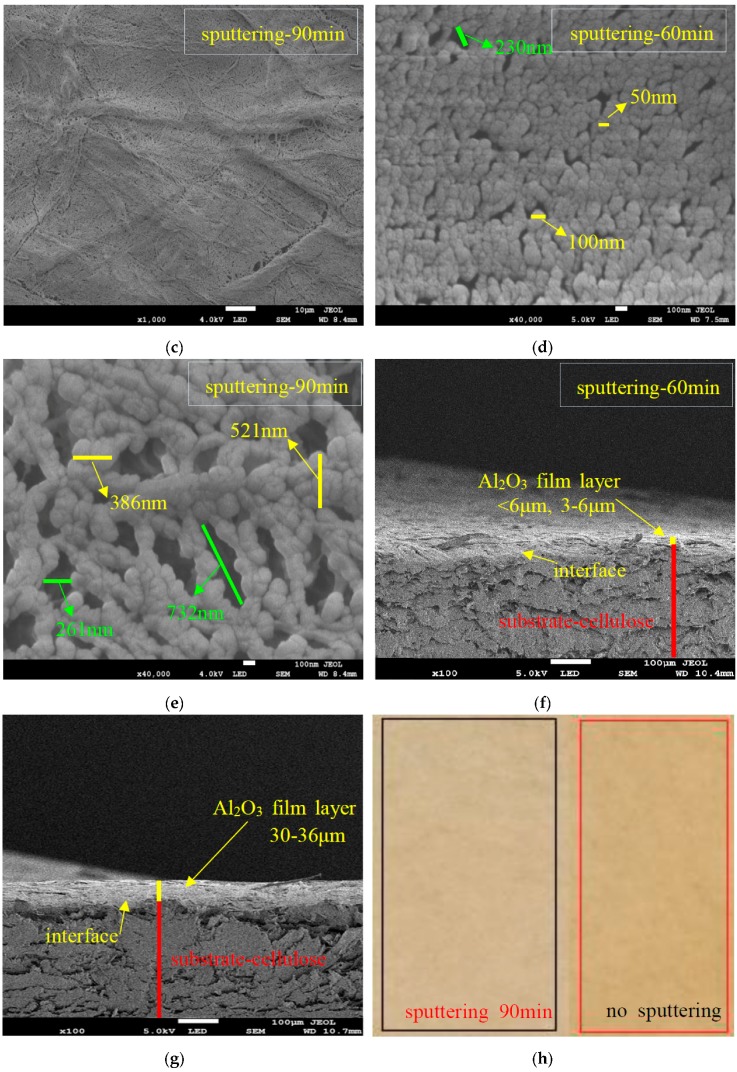
Scanning electron microscopy (SEM) images of the fresh pressboard surface at 1000× (**a**); pressboard surface prepared by reactive RF magnetron sputtering of Al target for 60 min at 1000× (**b**); 90 min at 1000× (**c**); 60 min at 40,000× (**d**); 90 min at 40,000× (**e**); the side elevation appearance of pressboard sputtered 60 min at 100× (**f**) and 90 min at 100× (**g**); the sputtered and fresh samples (**h**).

The SEM images for untreated pressboard and the pressboard surface prepared by magnetron sputtering of the Al target for 60 and 90 min are illustrated in Figure 3. It is evident that the structure of the surface obtained with sputtering (Figure 3b,c) changed. Figure 3a shows the SEM image for the untreated pressboard surface at 1000× magnification. The cellulose fibres were tied together and packed closely. There were some tiny holes where the fibres criss-cross each other. The pressboard surface with magnetron sputtering treatment for 60 min (Figure 3b) illustrates the formation of a thin film that deposited on the fibre surface. A significant change in the surface was observed for pressboard that had been sputtered for 90 min (Figure 3c). It was obvious that a thin film was deposited on the fibre surface. Figure 3d,e show the surface morphology of the pressboard sputtered for 60 and 90 min at 40,000× magnification, respectively. There was a dense, uniform distribution of tightly arranged particles deposited on the surface of the fibre sputtered 60 min (Figure 3d). Each particle was clearly seen, and the particle size of most particles was less than 100 nm in diameter. For the pressboard surface sputtered for 90 min, as shown in Figure 3e, the phenomenon of particle agglomeration occurred on the surface: the particles became larger, and bigger holes appeared compared to Figure 3d. As presented in Figure 3e, the particles accumulated together and continued to grow thicker and longer. They were arranged irregularly and overlapped with each other. The agglomerated particles were several hundred nanometres in diameter and at the micrometre level in length. The particle size of the agglomerated particles for 90 min was larger than that of particles sputtered for 60 min (Figure 3d,e).

Because of the non-metallic, non-rigid and rough properties of the fibre, its cross-sectional morphology was very difficult to measure. The side elevation appearance of the pressboard sputtered for 60 and 90 min is shown in Figure 3f,g. For sample sputtered 60 min, the thickness of the deposited layer was approximately 3–6 μm. The thickness of the film increased as the sputtering time was extended [27,32,33]. For sample sputtered 90 min, the thickness of the deposited layer was approximately 30–36 μm. The thickness shown in Figure 3 is the “appearance thickness”, not actual thickness. This is because there was a wide range of blending area for the Al_2_O_3_ and cellulose material at the interface between Al_2_O_3_ film and cellulose, due to the high roughness of the pressboard surface and the deep positon of the Al_2_O_3_ entered into the cellulose during sputtering. The pressboard is a loose structure of fibres, which is different from the dense structure of the metal or glass. This allows the sputtering material to enter the deep positon of the cellulose substrate easily. Compared with literature [37,38], the “appearance deposition rate” for the deposition layer was higher here, at a rate of about 0.90 μm/min. It was found that the wide range of blending area for the Al_2_O_3_ and cellulose material contributed a lot to the “appearance thickness” and “appearance deposition rate”. It is difficult to make accurate measurement of thickness due to the reason above. Besides, the interface phenomenon shown in Figure 3f,g indicated that the thin nano-structure layer attached to the fibre was successfully constructed.

**Figure 4:**
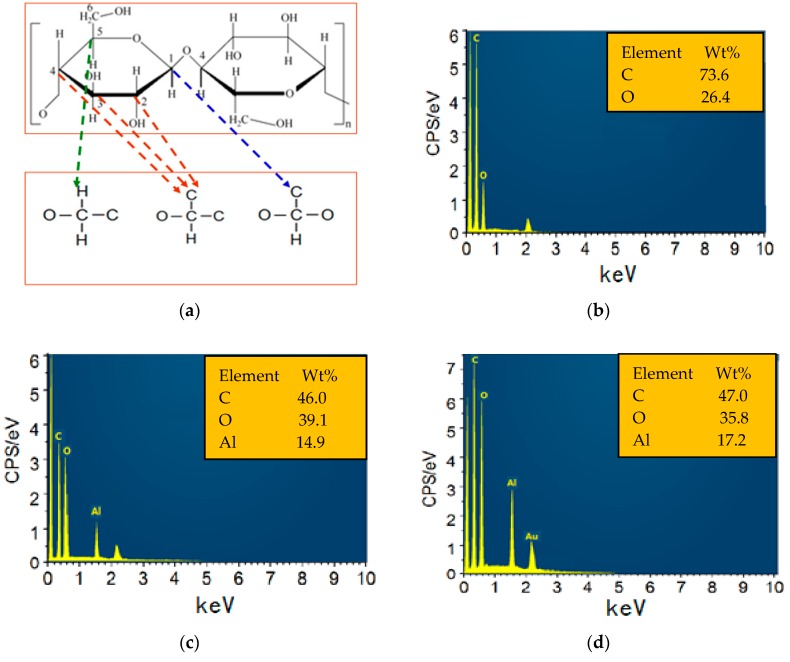
Chemical structure of the fibre (**a**); Energy dispersive spectrometer (EDS) of the fresh pressboard surface (**b**); EDS of the pressboard surface sputtered 60 min (**c**) and 90 min (**d**).

The chemical composition of the sputtered film was analysed using EDS. Figure 4a illustrates the chemical structure of cellulose. Cellulose consists of linear, polymeric chains of cyclic, β-d-glucopyranose units that are composed of C, H and O elements [39,40]. For the EDS spectrum of the cellulose insulation pressboard surface without sputtering shown in Figure 4b, only the elements C and O were observed, and Figure 4c,d revealed the presence of C, O, and Al on the pressboard surface that was prepared by reactive RF magnetron sputtering of the Al target for 60 and 90 min. The appearance of Al indicated that the aluminium oxide film was successfully fabricated on the fibre surface by reactive RF sputtering (O_2_ is the reactive gas). The Al content increased from 14.9% to 17.2% as the sputtering time increased from 60 to 90 min, indicating considerable changes in the composition of the as-prepared surface.

### 3.2. XPS of the Sputtered Film

**Figure 5:**
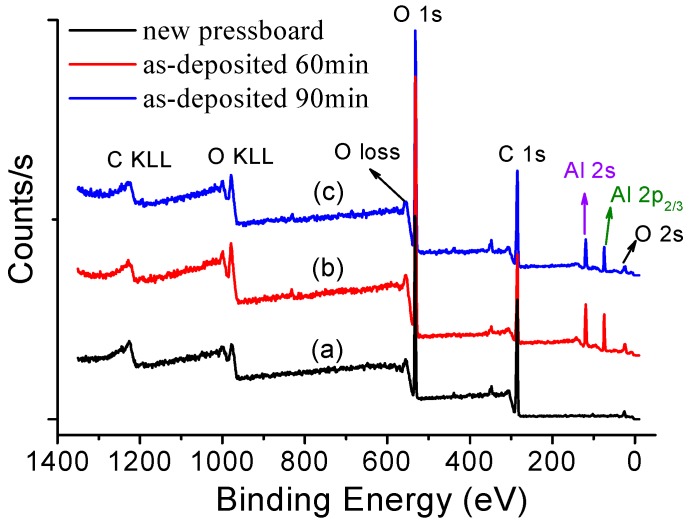
X-ray photoelectron spectroscopy (XPS) of fresh pressboard surface (**a**); pressboard surface sputtered for 60 min (**b**) and 90 min (**c**).

Figure 5 shows a comparison of the XPS survey spectra of the blank pressboard surface, the pressboard surface sputtered for 60 min, and the pressboard surface sputtered for 90 min. The peaks observed at the binding energies of approximately 74, 119, 285, 532, 979 and 1228 eV are associated with the chemical element states of O 2s, Al 2p, Al 2s, C 1s, O KLL, and C KLL, respectively. Compared with the fibre surface without sputtering, the new element peaks of Al 2p and Al 2s were detected from the sputtered fibre surface. The appearance of new Al element peaks in the XPS measurement also indicates that the aluminium oxide film has been fabricated on the pressboard surface.

**Figure 6:**
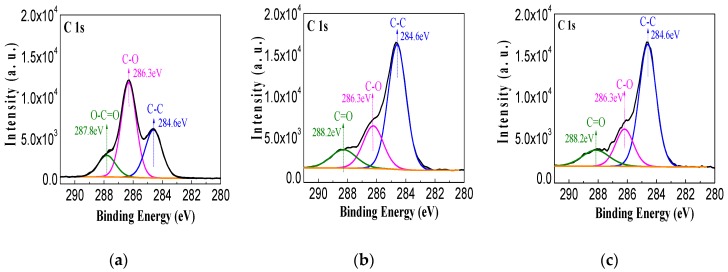

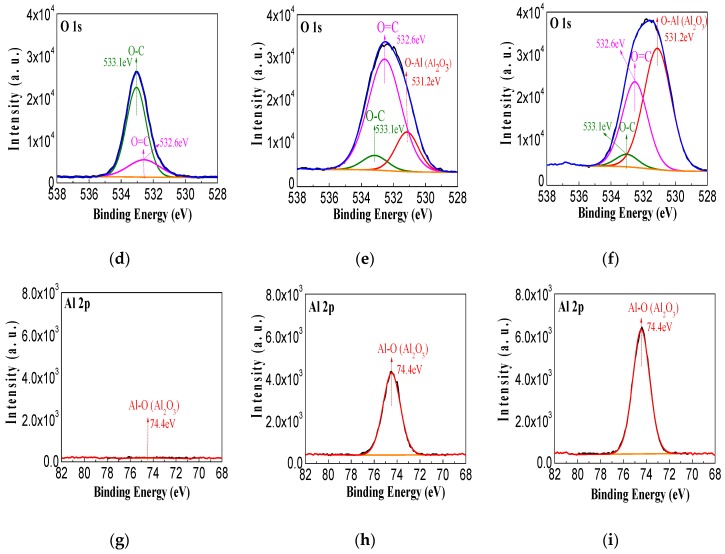
The C 1s, O 1s and Al 2p XPS spectra of fresh pressboard surface (**a**,**d**,**g**); pressboard surface sputtered for 60 min (**b**,**e**,**h**); and 90 min (**c**,**f**,**i**).

To elucidate the chemical state of the aluminium oxides film, resolution analysis of C 1s, O 1s and Al 2p was carried out. As presented in Figure 6a, the C 1s XPS spectra of the fresh pressboard surface and the sputtered fibre surface all had three peaks. The binding energy 284.6 eV was attributed to C–C, 286.3 eV is attributed to C–O and 288.3 eV was attributed to C=O [40,41]. The same peaks can be observed in Figure 6b,c, which are the C 1s XPS spectra of the pressboard surface sputtered for 60 and 90 min, respectively. Figure 6d–f clearly show that the O 1s XPS spectra of the sputtered pressboard surface were significantly different from those of the untreated pressboard. In addition to the intensity change of the C–O and C=O peaks, a new peak located at 531.2 eV originating from the O–Al bond can be seen for the sputtered pressboard surface. Figure 6g–i also show that a new peak appeared at 74.4 eV, originating from Al–O in the Al 2p XPS spectra of the sputtered pressboard surface. The intensity of the O–Al (Figure 6e,f) and Al–O (Figure 6h,i) peaks increase as the sputtering time increases. As reported in [41,42,43], the binding energy of O–Al (531.2 eV) shown in the O 1s spectra and the binding energy of Al–O (74.4 eV) shown in the Al 2p spectra were attributed to A1_2_O_3_. Moreover, the O–Al peak in Figure 6e,f and the Al–O peak in Figure 6h,i clearly indicated that there was only one peak and that no peak shifts occurred, which indicated that the Al had only one chemical state: Al^3+^. It is worth noting that no peak was observed around 73 eV, which corresponds to metallic Al. This indicates that the aluminium was completely oxidised during the reactive RF magnetic sputtering treatment. The binding energy difference between Al 2p and O 1s was 458 eV for samples sputtered for 60 and 90 min, which is similar to the literature [41]. Based on the XPS analysis results, the atomic content ratio of Al/O was 0.65, which is close to the stoichiometric composition of Al_2_O_3_ (Al/O = 0.66) [41,42,43]. Therefore, the thin film fabricated on the pressboard surface was Al_2_O_3_. The XPS peak for Al_2_O_3_ film at here is consistent with [44,45], where a thin Al_2_O_3_ layer was grown at room temperature and high temperature by atomic layer deposition.

### 3.3. XRD of the Sputtered Pressboard with Nano-Structured Al_2_O_3_ Film

**Figure 7:**
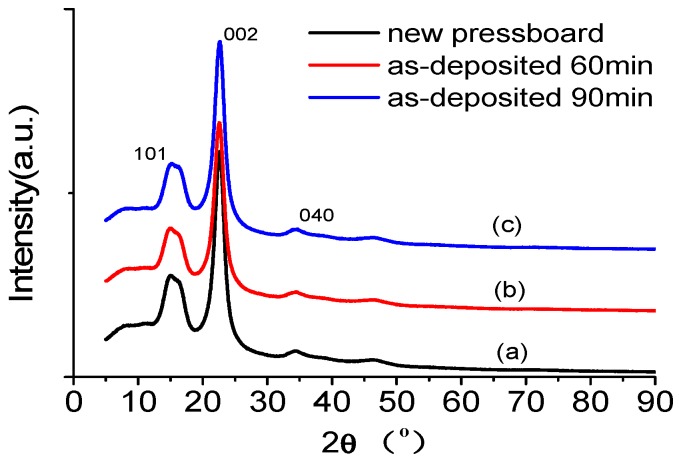
X-ray diffraction (XRD) of the fresh pressboard (**a**); pressboard surface fabricated Al_2_O_3_ film 60 min (**b**) and 90 min (**c**).

The XRD spectra of the blank pressboard and the pressboard surface fabricated with Al_2_O_3_ films are shown in Figure 7. The obvious diffraction peaks at 2θ = 14.93°, 2θ = 22.60° and 2θ = 34.85° were the characteristic phase (101), (002) and (040) diffraction peaks of cellulose I, respectively [39]. The broad and dispersive diffraction peaks show that the cellulose insulation pressboard had a two-phase (crystalline and amorphous) mixing structure. The correlation diffraction peak of Al_2_O_3_ was 30°–80° [33,43,46,47]. The diffraction peak of the α-Al_2_O_3_ (110) crystal surface was 2θ = 37.776°, and the diffraction peak of the α-Al_2_O_3_ (211) crystal surface was 2θ = 59.739°. The diffraction peak of the γ-Al_2_O_3_ (220) crystal surface was 2θ = 31.821°, and the diffraction peak of the γ-Al_2_O_3_ (311) crystal surface was 2θ = 37.5°. Figure 7a shows that there were no Al_2_O_3_ diffraction peaks for the untreated pressboard surface. However, the XRD results showed that there were also no Al_2_O_3_ diffraction peaks in the sputtered samples (Figure 7b,c). There was also a weak peak at 2θ = 46°–47°. According to the XRD standard spectroscopy of Al_2_O_3_, there was no peak for Al_2_O_3_ in the range of 46°–47°. As shown in reference [48], the peak between 46°–47° may be the sign of crystallisation for cellulose. The explanation for the lack of an XRD peak of Al_2_O_3_ is that Al_2_O_3_ exists in the films in amorphous form [43,46,47]. As reported in [41], Al_2_O_3_ is an amorphous structure when it is deposited below 500 °C. When the annealing temperature reaches 1200 °C, it is α-Al_2_O_3_. There is a mixture of α-Al_2_O_3_ and γ-Al_2_O_3_ when the annealing temperature is between 500 and 1200 °C. In this paper, the Al_2_O_3_ film is fabricated by reactive RF magnetic sputtering at room temperature; thus, the Al_2_O_3_ film is in the amorphous form.

### 3.4. Frequency Dielectric Property of the Sputtered Pressboard

**Figure 8:**
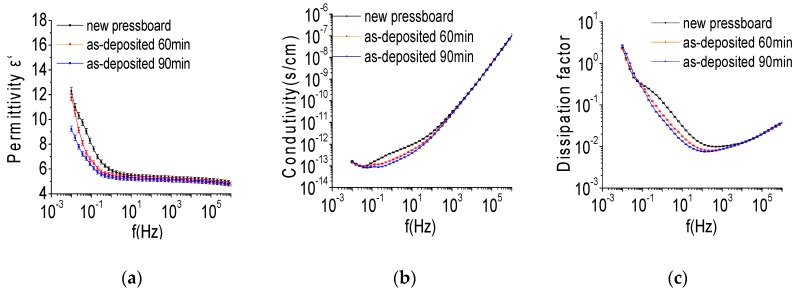
The $\varepsilon_{r}^{\prime }$ (**a**); conductivity (**b**) and tan δ (**c**) of the oil impregnated fresh pressboard, the oil impregnated pressboard surface sputtered Al_2_O_3_ film for 60 and 90 min.

Figure 8 shows the behaviours of the permittivity ($\varepsilon_{r}^{\prime }$), conductivity and the dissipation factor (tan δ) for the oil-impregnated fresh pressboard and the oil-impregnated pressboard surface sputtered with Al_2_O_3_ film for 60 and 90 min. The changing behaviour of $\varepsilon_{r}^{\prime }$, conductivity and tan δ of the pressboard surface coated by Al_2_O_3_ film was similar to the fresh pressboard. However, it is noteworthy that the pressboard surface coated by Al_2_O_3_ film had a lower $\varepsilon_{r}^{\prime }$, conductivity, and tan δ values in the lower frequency (<10^3^ Hz) region. This phenomenon is consistent with nano-filler dielectrics [12,15,17], although the conductivity and tan δ values in the frequency range of 10^−2^–10^−1^ Hz for the samples are nearly the same. The lower $\varepsilon_{r}^{\prime }$, conductivity and tan δ values for the sputtered pressboard provide good insulation properties, particularly the values at 50 or 60 Hz [15,17]. The lower $\varepsilon_{r}^{\prime }$ of the sputtered pressboard can reduce the difference of the dielectric constant between the pressboard and the insulating oil, and the lower conductivity and tan δ could make the pressboard less exothermic in field operation of the transformer. The results shown in Figure 8 indicate that the pressboard sputtered Al_2_O_3_ film with nano-structure benefits in its insulation performance $\varepsilon_{r}^{\prime }$, conductivity and tan δ). 

### 3.5. Space Charge Distribution in Sputtered Pressboard with Nano-Structured Al_2_O_3_ Film

**Figure 9:**
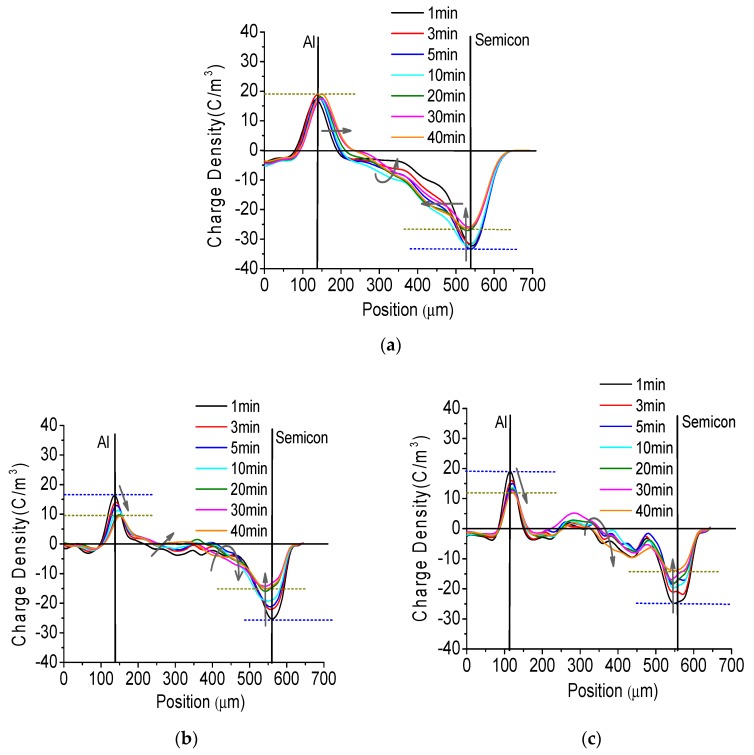
The space charge behavior of the oil impregnated fresh pressboard (**a**); the oil impregnated pressboard surface sputtered Al_2_O_3_ film for 60 min (**b**) and 90 min (**c**) under direct current (DC) 15 kV/mm.

**Figure 10:**
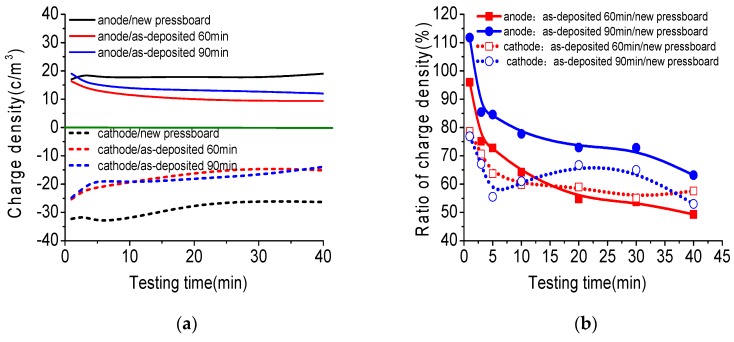

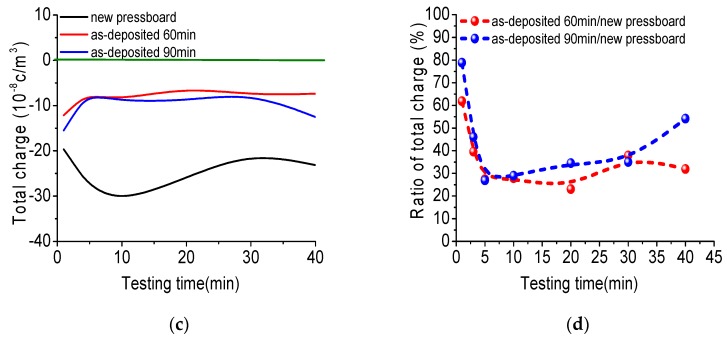
The charge density on the anode and cathode (**a**); the charge density ratio of treated samples to untreated samples (**b**); the total amount of charges trapped in the samples (**c**); and the charges amount ratio of treated samples to untreated samples (**d**) under DC 15 kV/mm.

To confirm that the nano-structured Al_2_O_3_ film performs the function of restraining charge injection and accumulation, the space charge distribution of fresh pressboard and pressboard sputtered Al_2_O_3_ films impregnated with new mineral oil is shown in Figure 9. In this experiment, the applied DC electric field strength was 15 kV/mm. The pressboard surface with only one side having sputtered Al_2_O_3_ film was attached to the cathode, and the other surface without treatment was attached to the anode. As the voltage was applied, the charge density on the anode/cathode and the total amount of charge trapped in the samples were determined, and are presented in Figure 10. 

The total amount of charge trapped in the samples was calculated based on Equation (1), where *ρ(x, t)* is the charge density at position *x* (*C/m^3^*), *S* is the electrode area (m^2^), and *d* is the thickness of the sample (m). The charge density ratio (*CDR*) of the sputtered pressboard (*SP*) to fresh pressboard (*FP*) was calculated based on Equation (2), where *SP_e_(t)* and *FP_e_(t)* is the charge density on the anode or the cathode for the sputtered pressboard and fresh pressboard, respectively, at time *t*. *CDR(t)* is the charge density ratio at time *t.* The charge amount ratio (*CAR*) for the sputtered pressboard (*SP*) to fresh pressboard (*FP*) is calculated according to Equation (3), where *CASP(t)* and *CAFP(t)* are the amount of charge trapped in the sputtered pressboard and fresh pressboard, respectively, at time *t*. *CAR(t)* is the charge amount ratio at time *t*.

(1) $$Q\left(t\right)=\int_{0}^{d}\rho \left(x,t\right)Sdx$$

(2) $$\mathrm{CDR}(t)=\frac{SP_{e}(t)}{FP_{e}(t)}\times 100\%$$

(3) $$\mathrm{CAR}(t)=\frac{CASP(t)}{CAFP(t)}\times 100\%$$

In Figure 9, the black vertical real line is the position of the anode and the cathode. The distance between the anode and the cathode is the thickness of the sample. The arrow’s direction represents the direction of charge movement. As shown in Figure 9, homo-charge injection occurred shortly after the voltage is applied for the fresh pressboard and pressboard sputtered by Al_2_O_3_ films. For the fresh pressboard sample (Figure 9a), the charge density lines of positive charge near the anode and of negative charge near the cathode both moved towards the inner side of the pressboard. A more obvious negative charge injection was observed, which caused the density of negative charges on the cathode to decrease with time until stability was reached, and the positive charges on the anode showed nearly no change after applying the voltage for 5 min (Figure 10a). In the middle part of the sample, there was mainly a negative charge. Its density increased when applying 0–10 min of voltage and then decreases with time, until approximate stability was reached after 30 min. The amount of charge trapped in the fresh pressboard first increased and then decreases until it was close to stability, as shown in Figure 10c.

**Figure 11:**
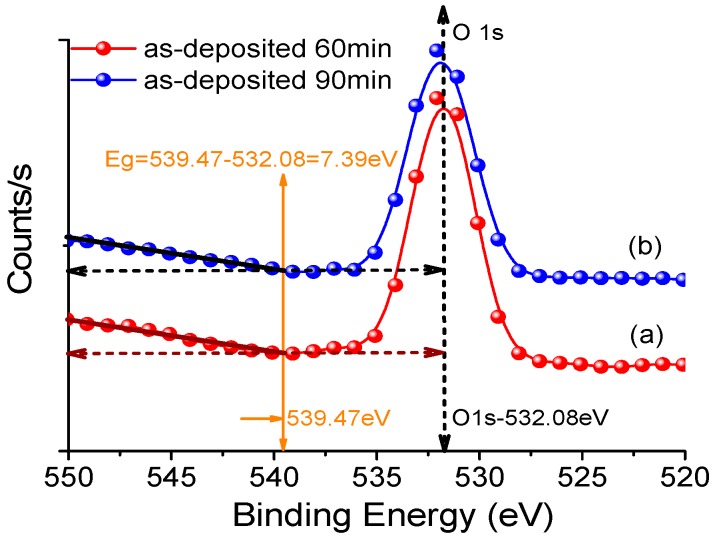
The band gap of Al_2_O_3_ from the O 1s peak obtained by high resolution XPS.

For the pressboard sputtered with Al_2_O_3_ film for 60 min shown in Figure 9b, it can be seen that the charge injection phenomenon was not obvious compared to that of the fresh pressboard. The densities of the negative charge on the cathode and the positive charge on the anode both decreased with time until stability was reached. Because the Al_2_O_3_ film was attached to the negative electrode surface, not many negative charges were trapped in the bulk of the sample, as observed for the fresh pressboard. The negative charge injection was effectively suppressed. The charge density on the anode and cathode (Figure 10a) and the amount of charge trapped in the pressboard sputtered with Al_2_O_3_ film for 60 min (Figure 10c) were much lower than those in the fresh pressboard during the process of applying voltage. It was particularly noteworthy from the charge density ratio result shown in Figure 11b that the charge density value on the anode and cathode of the pressboard sputtered for 60 min was only 49% and 57%, respectively, of the untreated pressboard when the charge injection becomes almost stable (applying the voltage for 40 min). Further, Figure 10d showed that the total amount of charge trapped in the pressboard sputtered for 60 min was only 31% of that of the untreated pressboard (applying the voltage for 40 min).

For the pressboard sputtered with Al_2_O_3_ film for 90 min shown in Figure 9c, because of the suppressed effect of charge caused by the Al_2_O_3_ film on the negative electrode side, the charge injection phenomenon was also not obvious compared to that of the untreated pressboard. However, it is slightly more significant than that of the pressboard sputtered with Al_2_O_3_ film for 60 min. It could be seen that many negative charges were trapped in the vicinity of the cathode and that positive charges accumulate in the middle part of the sample. The density of the charges on the anode and cathode and the amount of charge in the sample were both much lower than those of the untreated pressboard (Figure 10a,c). As shown in Figure 11b, the charge density on the anode and cathode of the coated pressboard 90 min is 63% and 53% of the untreated pressboard when the charge injection becomes stable (applying the voltage for 40 min). The total amount of charge trapped in the pressboard sputtered for 90 min was only 54% of the untreated pressboard as the voltage was applied for 40 min, as presented in Figure 10d.

## 4. Discussion

As shown in Figure 9 and Figure 10, the oil-impregnated sputtered pressboard presented an apparent space charge suppression effect. The charge density on the anode for the pressboard sputtered for 60 min was much less than that of the pressboard sputtered for 90 min (Figure 10a,b), and the charge density on the cathode for the pressboard sputtered for 60 min was not much different from that of the pressboard sputtered for 90 min (Figure 10a,b). The amount of charge trapped in the pressboard sputtered with Al_2_O_3_ film for 60 min was less than that in the pressboard sputtered with Al_2_O_3_ film for 90 min (Figure 10c,d). Therefore, compared with the pressboard sputtered with Al_2_O_3_ film for 90 min, the pressboard sputtered with Al_2_O_3_ film for 60 min had a better space charge suppression effect. 

Figure 3, Figure 4, Figure 5, Figure 6 and Figure 7 confirm that the significant suppression of the charge injection and accumulation obviously resulted from the chemical composition variation and structural alteration of the surface layer because of the sputtered nanostructured Al_2_O_3_ film. At present, space charge suppression mechanisms focus mainly on nano-dielectrics (nano-filler dielectrics, in which the nano-fillers are usually 1 to 100 nm in size). J. Keith Nelson et al. [49] proposed that the nanoparticles could decrease the trapped energy, thereby increasing the thermal detrapping probability and the carrier mobility, ultimately leading to less space charge accumulating in the bulk of the sample. T. Tanaka [50,51] proposed that the conductivity of the interaction zone between the nanoparticle and the polymer matrix is much higher, which leads the carriers to prefer to conduct through the interaction zone, thereby causing the space charge suppression. In addition, the contact potential barrier might be affected by the interaction zone. An increase in the contact potential barrier will decrease the accumulated space charge in nano-dielectrics. Takada et al. [52] noted that trap centres near the interface between the electrode and the nano-dielectrics can capture the injected charges, which could distort the electric field and decrease the charge injection. However, only a few studies have focussed on the nanostructured film/electrodes interface properties to explain the charge injection effect [50]. Milliere et al. investigated the efficient barrier effect of the AgNPs/plasma polymer stack on the charging behaviour of polyethylene [52]. Tailoring the surface of LDPE films with an AgNPs/SiOxCy:H stack provides a viable solution for space charge moderation. AgNPs can accommodate positive and negative charges because of their barrier effect for both polarities. The nanoparticles can increase the injection barrier height between the electrode and the material, which plays a more important role in the suppression of space charge [53]. 

Based on the above analysis, we can deduce that the Al_2_O_3_ nano-structured film sputtered on the fibre surface could act as a functional barrier layer for charge injection. The band gap energy (Eg) of Al_2_O_3_ can be derived from the energy difference between the elastic peak energy (E O1s) and the onset of inelastic loss energy (E loss): Eg = E loss − E O1s [41,54,55]. As shown in Figure 11, the high resolution O 1s spectrum shows that the energy of the elastic peak (E O1s) is 532.08 eV. The energy of onset of inelastic losses (E loss), which is measured from the O 1s spectrum by intersecting the linear-fit line and the background zero level, is 539.47 eV. Therefore, the obtained band gap energy (Eg) of Al_2_O_3_ is 7.39 eV. The measured band gap energy of Al_2_O_3_ is lower than the reported value of Al_2_O_3_ (8.8 eV) [54], which is consistent with the results shown in [41]. This may be attributed to the defect structure of Al_2_O_3_, and requires further investigation [39,54]. The nano-structured Al_2_O_3_ film introduces a new trap band that is helpful for charge inhibition.

**Figure 12:**
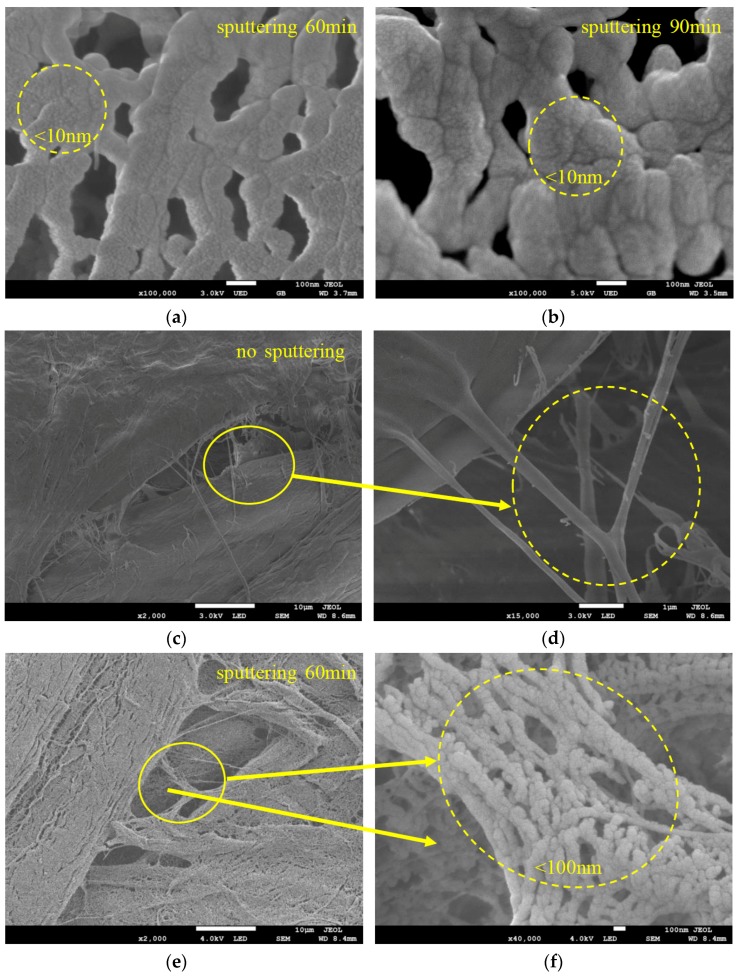
SEM images of the pressboard surface sputtered 60 min at 100,000× (**a**), 90 min at 100,000× (**b**), fresh pressboard surface at 2000× (**c**) and 15,000× (**d**), pressboard surface sputtered 60 min at 2000× (**e**) and 40,000× (**f**).

In addition, compared to the Al_2_O_3_ nano-structured film sputtered for 90 min, the Al_2_O_3_ nano-structured film sputtered for 60 min is much denser (Figure 3d,e), and the nano-Al_2_O_3_ particles have smaller particle size (<100 nm). It is particularly emphasised that when the image is magnified by 10,000 times, a large number of particles with much smaller particle sizes (<10 nm) cling to the larger particles (>50 nm), as shown in Figure 12a,b, where the circle is marked. Compared with pressboard sputtered for 90 min, more ultra-small particles (<10 nm) grow on the surface of the larger nanoparticles in pressboard sputtered for 60 min. If a larger nanoparticle (>50 nm) is compared to a Duchesnea (mock-strawberry), these ultra-small nanoparticles (<10 nm) are similar to the tiny particles embedded on the surface of the Duchesnea. These nanoparticles, especially the ultra-small nanoparticles (<10 nm), may lead to better suppression of charge injection and accumulation. 

In addition, the injection and accumulation of space charge is closely related to the defects of the sample. Figure 12c,d show that the surface of the fresh pressboard is very rough, with many tiny voids and fibres distributed randomly on the surface. These would lead to greater charge generation and injection. However, when the fibre surface is being sputtered by Al_2_O_3_ nano-structured film, the tiny voids and fibres would be covered by the nano-Al_2_O_3_. Figure 12e,f show the nano-Al_2_O_3_ particles attached on the fibre and on the bottom of the tiny voids. The superior properties of nanoparticles have the function of weakening the physical defects to a great extent.

## 5. Conclusions

The nano-structured Al_2_O_3_ film was successfully fabricated on a cellulose insulation pressboard surface by reactive RF magnetron sputtering. The SEM results showed that there are dense, uniformly distributed and tightly arranged Al_2_O_3_ particles less than 100 nm in diameter deposited on the surface of fibres sputtered for 60 min. Particle agglomeration occurred on the surface when the sputtering time was increased, and the agglomerated particles were several hundred nanometres in diameter and at the micrometre level in length. It is of great importance that larger amounts of ultra-small particles (<10 nm) grow on the surface of the larger nanoparticles. XPS analysis showed that new peaks located at 74.4 and 531.2 eV appeared for the sputtered pressboard, and that the atomic content ratio of Al/O was 0.65, which confirmed that the deposited film is Al_2_O_3_. The XRD results showed that the nano-structured Al_2_O_3_ was in the amorphous form. 

The cellulose insulation pressboard surface sputtered by Al_2_O_3_ film had lower ε’r, conductivity and tan δ values in the lower frequency (<10^3^ Hz) region, which benefits its insulation performance. Compared with the pressboard sputtered with Al_2_O_3_ film for 90 min, the pressboard sputtered with Al_2_O_3_ film for 60 min had a better space charge suppression effect. At DC 15 kV/mm, the charge density value on the anode and cathode of the pressboard sputtered for 60 min was only 49% and 57% that of the untreated pressboard, and the total amount of charge trapped in the sputtered pressboard was less than 50% that of the untreated pressboard. The nano-structured Al_2_O_3_ film sputtered on the fibre surface could act as a functional barrier layer for suppression of the charge injection, and provide the function to weaken physical defects, especially the ultra-small nanoparticles (<10 nm). The obtained band gap energy (Eg) of Al_2_O_3_ was 7.39 eV.

The present study hopefully provides a novel way to suppress space charge by designing a structured surface on the cellulose insulation pressboard, which has a potential application for use in the high-performance insulation material used in HVDC equipment. Future work is needed to focus on the analysis of the interfacial properties of the nano-structured Al_2_O_3_ film and the electrode, as well as the suppression effect of the double-sided coated Al_2_O_3_ film.
